# Editorial

**Published:** 1872-01-15

**Authors:** 


### MEDICAL EXAMINER.

*A Semi-Monthly Journal of Medical Sciences.*

EDITED BY

N. S. DAVIS, M. D., AND F. H. DAVIS, M. D.

Chicago, January 15th, 1872.

### EDITORIAL.

CHICAGO MEDICAL COLLEGE MUSEUM.—During the past year many valuable additions have been made to this museum; and among them is a complete and elegantly prepared human skeleton, donated by the executors of the estate of the late Dr. James Cooper, of New Castle, Delaware. It is prized more highly as a memento, it having been prepared by Dr. Cooper during his pupilage, and left as a part of his estate after a long and most useful professional life.

RUSH MEDICAL COLLEGE COMMENCEMENT.—The twenty-ninth annual commencement exercises of the Rush Medical College took place in the Lecture Room of the Michigan avenue Baptist Church, on the evening of the 17th of January, 1872. The graduating class numbered seventy-seven, and the exercises were in the usual form. The charge to the graduates was delivered by Prof. J. W. Freer, President of the College. As the present term commenced on the last Wednesday in September, it will be seen that its entire length has been a trifle more than three months and a half. When we remember that the founders of the first medical school in America, one hundred years since, thought six months the minimum length of time necessary for a full course of medical instruction at that time, when the facts and items to be taught were hardly half what they are now, we cannot fail to see that the cause of medical education has made *progress.* For any young man to give an adequate review of the essential branches of medical science as they now exist in sixteen weeks, he must certainly go it with a *Rush!* But we will not complain. On the contrary, when our neighbors get their new edifice up, Phœnix-like, from the ashes of the former one, we trust they will not only carry out a longer term, but adopt the more rational graded system of instruction.

MOST of our medical and scientific journals, burned out in the great fire, have again resumed publication. *The Pharmacist*, *The Medical Times*, Eclectic, and the *The Medical Investigator*, Homœpathic, have appeared in their old style and form. The *Lens*, a new journal of microscopy and allied natural sciences, published by the Illinois State Microscopical Society, has also reappeared. The first number was destroyed in the printing office when just ready for delivery. This number has been replaced, and the issues will hereafter appear regularly each month.

The Chicago *Medical Journal* has just been issued, the Oct., Nov. and Dec. numbers together.

BLISS & TORREY, successors to the old and well-known firm of Bliss & Sharp, in their surgical instrument department, have opened a new store at No. 25 Market street, where they have a complete assortment of Tieman's celebrated surgical instruments, and physician's goods generally.

AMERICAN JOURNAL OBSTETRICS AND DISEASES OF WOMEN AND CHILDREN.—This valuable quarterly is now published by Wm. Baldwin & Co., 21 Park Row, New York. They offer to send a sample copy to any physician for 50 cents, one-third of the regular subscription price.

E. H. SARGENT, DRUGGIST.—We take pleasure in calling the attention of our readers to the fact that our old friend E. H. Sargent has become fully established in his new store, on the corner of Wabash avenue and Sixteenth street. For skill as a pharmaceutist and integrity in the dispensing of pure and reliable medicines, we can fully commend him to the patronage of the profession both in the city and country.

